# Bile acids affect intestinal barrier function through FXR and TGR5

**DOI:** 10.3389/fmed.2025.1607899

**Published:** 2025-07-07

**Authors:** Guangyao Song, Yuxiao Xie, Lanlan Yi, Wenjie Cheng, Huijin Jia, Wenzhe Shi, Qiwei Liu, Ligui Fang, Shiqi Xue, Dan Liu, Junhong Zhu, Sumei Zhao

**Affiliations:** ^1^College of Animal Science and Technology, Yunnan Agricultural University, Kunming, China; ^2^College of Biology and Agriculture, Zunyi Normal University, Zunyi, China

**Keywords:** bile acids, bile acid receptors, FXR, TGR5, intestinal barrier

## Abstract

Bile acids play a dual role by aiding lipid absorption and acting as signaling molecules by interacting with various receptors. Bile acids are perpetually recycled via enterohepatic circulation and are biotransformation by gut microbiota, making bile acid metabolism a critical regulator of intestinal homeostasis. The intestinal epithelium prominently expresses two key bile acid receptors - the farnesoid X receptor (FXR) and G protein-coupled bile acid receptor 1 (TGR5) - which play indispensable roles in maintaining bile acid homeostasis and intestinal barrier function. Due to the abundant expression of bile acid receptors and the importance of the intestine in preventing pathogen invasion, researchers are increasingly focused on the function of bile acids in this system. This article focuses on the effect of bile acids and their receptors, FXR and the TGR5, in modulating intestinal barrier function.

## 1 Introduction

The core structure of bile acids (BAs) consists of 17 carbon atoms arranged in three hexane rings and one pentane ring (1). BAs possess both hydrophilic and hydrophobic regions, enabling them to form mixed micelles with lipids and their digestive products. This character allows cholesterol and other lipophilic substances to dissolve in bile, promoting the emulsification and absorption of fats and fat-soluble vitamins, while also regulating cholesterol stability (2, 3). Furthermore, BAs are recognized as important signaling molecules that play a role in regulating lipid metabolism, glucose metabolism, and energy metabolism, exhibiting hormone-like functions (4). Moreover, bile acids play a crucial role in the intestinal barrier. Different bile acids have varying effects on intestinal epithelial cells. Lithocholic acid (LCA) and deoxycholic acid (DCA) may promote epithelial renewal by inducing programmed cell death, while ursodeoxycholic acid (UDCA) may protect epithelial cells (5–7). The role of BAs is also crucial in inflammatory bowel diseases (IBDs). Research has shown that patients with Crohn’s disease have a smaller bile acid pool, and the ratio of glycine to taurine complexes significantly increases. The decrease in bile acid pool size is related to disease activity (8). The deficiency of secondary BAs produced by gut microbiota can promote intestinal inflammation, while DCA and LCA can alleviate inflammation in mouse colitis models (9).

Given the high expression levels of bile acid receptors and the gut’s critical role in preventing pathogen invasion, the significance of BAs in the gut garners increasing attention from researchers (10). This review aims to elucidate the functions of bile acid metabolism and its intricate relationship with the intestinal barrier, detailing the regulatory mechanisms by which BAs and their primary receptors, FXR and TGR5, influence the mechanical, mucosal, microbial, and immune barriers of the intestine.

## 2 BAs metabolism

In the liver, BAs are synthesized from cholesterol through the action of at least 17 enzymes (11, 12). There are two primary pathways for BA synthesis: the classical (neutral) pathway and the alternative (acidic) pathway. The classical pathway is facilitated by cholesterol 7 α-hydroxylase (CYP7A1), resulting in the production of cholic acid (CA) and chenodeoxycholic acid (CDCA). Conversely, the alternative pathway is mediated by sterol 27-hydroxylase (CYP27A1), which exclusively produces CDCA. The BAs generated through these pathways are referred to as primary BAs (13). In the liver, primary BAs can conjugate with glycine or taurine at the C-24 position to form conjugated BAs (14).

Bile acids synthesized in the liver are secreted into the bile duct and stored in the gallbladder, facilitated by the bile salt export pump (BSEP) and the phosphatidylcholine transporter (ABCB4). Upon food intake, cholecystokinin released from the duodenum triggers gallbladder contraction, leading to the release of bile into the intestine (15–17). As BAs traverse the intestine, a small fraction of unbound BAs is reabsorbed through passive diffusion, while the majority is actively absorbed via the apical sodium-dependent bile acid transporter (ASBT) in the distal ileum, subsequently entering the liver through the portal vein (13, 18). The reabsorbed and primary BAs undergo further processing in the liver before being secreted back into the gallbladder and re-entering the intestine, thereby establishing the enterohepatic circulation of BAs.

A portion of the BAs that are not absorbed is converted into secondary BAs through microbial action in the distal ileum and colon. For instance, microbe with bile salt hydrolase (BSH) activity deconjugate CA and CDCA, yielding unconjugated primary BAs, which are then transformed into secondary BAs via microbial 7-dehydroxylation (19, 20). Deoxycholic acid (DCA), derived from CA, is reabsorbed and returned to the liver from the colon, while lithocholic acid (LCA), produced from CDCA, poses a health risk; only a small amount of LCA is transported to the liver for detoxification through sulfation before entering the bile, with the majority excreted in feces (21).

The bile acid pool contains approximately 3 g of BAs, which circulate 6–15 times daily, with about 0.2–0.5 g excreted in feces (22). This loss of BAs is compensated by the de novo synthesis, ensuring the stability of the bile acid pool (23). Furthermore, the excretion of BAs in feces serves as a primary mechanism for the body to eliminate cholesterol (24).

## 3 BA receptors

Bile acid receptors can be classified into two main types: nuclear receptors and membrane receptors. The nuclear receptors encompass FXR (25, 26), vitamin D receptor (VDR) (27), pregnane X receptor (PXR) (28), and constitutive androstane receptor (CAR) (29). In contrast, the membrane receptors include the G protein-coupled receptor 1 (TGR5) (7) and sphingosine 1-phosphate receptor 2 (S1PR2) (30). Among these, the nuclear receptor FXR and the membrane receptor TGR5 are the most thoroughly investigated receptors to date.

### 3.1 The nuclear receptor FXR

**FIGURE 1:**
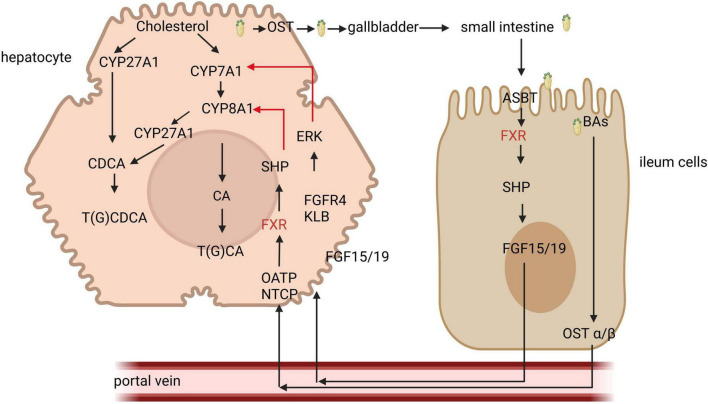
The role of farnesoid X receptor (FXR) signal in bile acid regulation. Bile acids (Bas) are synthesized from cholesterol via both classical and alternative pathways. The classical pathway is regulated by the key enzyme CYP7A1, whereas the alternative pathway is facilitated by CYP27A1. The primary BAs produced are stored in the gallbladder. Upon ingestion of food, the gallbladder contracts, releasing BAs into the intestine. These BAs are then absorbed in the distal ileum and transported back to the liver through the portal vein. Within liver cells, BAs activate FXR receptors, which in turn promote the expression of small hetero-dimer partner (SHP), resulting in the inhibition of CYP8A1. Additionally, BAs that enter intestinal cells activate FXR, leading to the secretion of FGF15/19, which subsequently travels to the liver via the portal vein to inhibit CYP7A1, thereby reducing bile acid synthesis. NTCP, sodium taurocholate co-transporting polypeptide; FGF, fibroblast growth factor; ERK; extracellular signal-regulated kinases.

The nuclear receptor FXR was the first bile acid receptor to be identified (25) and is predominantly expressed in tissues that participate in the hepatic-intestinal circulation of BAs (12). FXR can be activated by BAs to exert its biological effects, although its activation potential varies among different BAs. Hydrophilic BAs, such as UDCA does not activate FXR and Muricholic acids (MCA) are known to antagonize FXR, whereas hydrophobic BAs activate FXR in the following order: CDCA > LCA = DCA > CA (31, 32). Upon activation, FXR regulates the expression of various genes either as monomers or in heterodimeric complexes by binding to the retinoic acid X receptor (RXR) on DNA (33, 34). FXR serves as a sensor for intracellular bile acid levels and plays a crucial role in maintaining bile acid homeostasis (Figure 1).

In the intestine, BAs enter intestinal cells through the action of the apical sodium-dependent bile acid transporter (ASBT) and subsequently bind to the ileal bile acid binding protein (IBABP), facilitating their transport from the apical surface to the basolateral membrane. With the assistance of the organic solute transporter (OST) and organic anion transporting polypeptides (OATP), BAs then enter the portal vein and are transported to the liver. In the liver, they bind to the fibroblast growth factor receptor 4 (FGFR4) and β Klotho (KLB), leading to the downregulation of the rate-limiting enzyme CYP7A1 in the classical pathway of the extracellular signal regulated kinase (ERK), thereby inhibiting bile acid synthesis (35).

In liver cells, BAs stimulate FXR, which induces the expression of the small hetero-dimer partner (SHP) and inhibits the expression of the key enzyme CYP8B1 (sterol 12α-hydroxylase) involved in bile acid synthesis, thus regulating bile acid metabolism by modulating genes associated with bile acid secretion (36). The activation of FXR can also improve liver disease. In patients with non-alcoholic fatty liver disease (NAFLD) or in mouse models, treatment with FXR agonist obeticholic acid (OCA) produced a series of related liver effects, reducing triglycerides (TAGs) and inflammation, and alleviating steatohepatitis and liver fibrosis (37). FXR also influences molecules related to bile acid transport, with IBABP exhibiting a high affinity for BAs. In intestinal epithelial cells, FXR can regulate bile acid homeostasis by modulating the IBABP gene (38). Additionally, FXR is implicated in intestinal immune regulation and the maintenance of intestinal barrier function (39, 40). Activation of FXR has been shown to mitigate inflammation in animal models of inflammatory bowel disease (IBD), alleviate colitis symptoms, protect the intestinal epithelial barrier, and reduce the loss of goblet cells (41).

### 3.2 The membrane receptor TGR5

**FIGURE 2:**
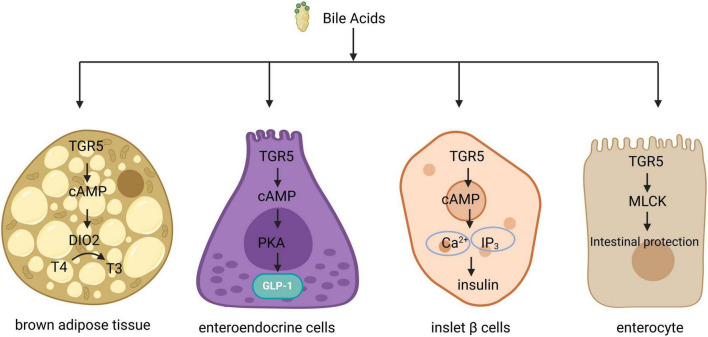
Signal transduction of TGR5 in different organizations. In brown adipose tissue, TGR5 activation facilitates the conversion of thyroid hormone T4 to T3 by inducing type 2 deiodinase, thereby enhancing energy metabolism. In intestinal endocrine cells, TGR5 activation triggers the activation of protein kinase A (PKA), which subsequently promotes the secretion of glucagon-like peptide-1 (GLP-1). In pancreatic beta cells, TGR5 activation results in elevated levels of intracellular cyclic adenosine monophosphate (cAMP) and Ca2+, leading to an increase in insulin secretion. Additionally, in intestinal epithelial cells, TGR5 activation stimulates the MLCK signaling pathway, contributing to epithelial protection. TGR5, G protein-coupled bile acid receptor 1.

G protein-coupled bile acid receptor 1 is a significant bile acid membrane receptor that is predominantly expressed in the gallbladder, ileum, and colon (42–44). In the liver, TGR5 regulates microcirculation, inflammation, regeneration, bile secretion and proliferation, as well as gallbladder filling (45). TGR5 has also been identified as a negative regulator of liver inflammation. Mice lacking TGR5 are more susceptible to liver injury after intraperitoneal injection of lipopolysaccharide, leading to increased levels of inflammatory cytokines and enhanced liver cell apoptosis (46). The potency of BAs in activating TGR5 follows the hierarchy: LCA > DCA > CDCA > CA (47). Upon binding with BAs, TGR5 triggers the release of a complex comprising G proteins—αs, β, and γ. This interaction facilitates the exchange of GDP for GTP within the G protein complex, resulting in the dissociation of the complex and the formation of G protein - αs and β - γ dimers. The G protein - αs subunit activates adenylate cyclase, which in turn promotes the synthesis of cyclic adenosine monophosphate (cAMP) and the activation of protein kinase A (PKA). This cascade initiates downstream signaling pathways, while cAMP production also influences energy and glucose metabolism (48). BAs regulate metabolic processes differently across various tissue types (Figure 2). For instance, the elevation of cAMP levels in brown adipocytes, induced by bile acid treatment, enhances the activity and oxygen consumption of type 2 iodothyronine deiodinase (D2), thereby contributing to the regulation of energy homeostasis (49). Additionally, glucagon-like peptide-1 (GLP-1) can lower blood glucose levels, and BAs in the intestine stimulate GLP-1 secretion from intestinal endo-crine cells via TGR5, thus impacting glucose metabolism (50). Furthermore, TGR5 activation in pancreatic beta cells promotes insulin secretion through the G-αs/cAMP/Ca2+ signaling pathway (51). TGR5 also plays a crucial role in maintaining intestinal barrier function; in intestinal cells, TGR5 activation by BAs stimulates myosin light chain kinase (MLCK) signaling, thereby enhancing intestinal barrier protection (52) (Figure 2). Moreover, TGR5 is involved in the regulation of bile acid metabolism, as it modulates the expression of cholesterol 12α-hydroxylase (CYP8B1), a key enzyme in bile acid synthesis, leading to a reduction in the proportion of 12α-hydroxy BAs in the bile acid pool (53).

## 4 The role of BAs in the intestinal barrier

### 4.1 Mechanical barriers

The intestinal epithelium consists of a single layer of columnar cells approximately 20 μm thick, and the epithelial layer not only comprises intestinal epithelial cells but also includes secretory cells such as goblet cells, Paneth cells, and intestinal endocrine cells (54). Within the intact epithelial layer, tight junctions (TJs) and adherens junctions (AJs) serve as critical cellular connections that contribute to the mechanical barrier of the intestine (55, 56). TJs are intercellular connections located at the apical region of cell contact, playing a vital role in determining epithelial permeability and regulating paracellular transport pathways (57). They are essential connections between epithelial cells, formed by proteins such as claudins, occludin, and junctional adhesion molecules (JAMs), which effectively prevent the invasion of intestinal bacteria and toxins (54). Alterations in the expression, post-translational modifications, localization, or activity of tight junction proteins or their regulatory factors can influence the permeability to larger molecules (58). Damage to epithelial cells disrupts the intestinal barrier, resulting in a loss of barrier function and allowing substances from the intestinal lumen to penetrate the submucosal layer (54). AJs, located on the lateral membranes of epithelial cells beneath the TJs, primarily function to maintain intercellular contact, cell polarity, motility, and proliferation (56). The structure of adherens junctions mainly consists of transmembrane glycoproteins from the classical cadherin superfamily (such as E-cadherin) and members of the catenin family (including p120 catenin, β-catenin, and α-catenin), which collectively regulate the formation, maintenance, and functionality of these connections (59). Additionally, adherens junctions provide mechanical strength to adjacent epithelial cells (60).

**FIGURE 3:**
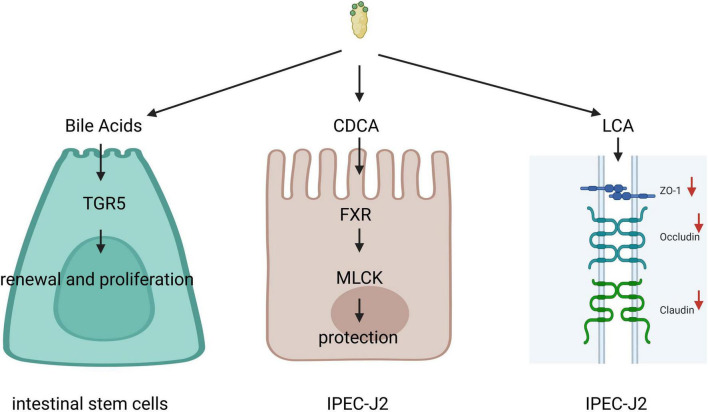
Bile acids (Bas) and their receptors are intricately linked to the mechanical barrier function of the intestine. Bile acids can promote epithelial regeneration by activating the membrane receptor G protein-coupled bile acid receptor 1 (TGR5) in intestinal stem cells (ISCs), chenodeoxycholic acid (CDCA) can protect intestinal epithelial barrier function through the farnesoid X receptor (FXR)-myosin light chain kinase (MLCK) pathway. Lithocholic acid (LCA) can downregulate the expression of tight junction proteins and genes in IPEC-J2.

Bile acids and their receptors are intricately linked to the mechanical barrier function of the intestine (Figure 3). Research indicates that BAs can enhance epithelial regeneration by activating the membrane receptor TGR5 in intestinal stem cells (ISCs). It has been demonstrated that the release of endogenous BAs in the intestinal lumen is sufficient to coordinate the renewal and proliferation of ISCs (7). Chenodeoxycholic acid (CDCA) has been shown to promote the proliferation of piglet jejunal epithelial cells (IPEC-J2), accelerate cell cycle progression in the S and G2/M phases, improve mitochondrial function, reduce intracellular reactive oxygen species (ROS) production in IPEC-J2 cells (61), thereby exerting a beneficial effect on these cells and protecting intestinal epithelial barrier function from lipopolysaccharide-induced damage via the FXR-MLCK pathway (62). In vitro, studies have revealed that CDCA, deoxycholic acid (DCA), and cholic acid (CA) can induce a transient reduction in transepithelial resistance in Caco-2 cells and modulate intestinal permeability by promoting the self-phosphorylation of epidermal growth factor (EGF) receptors, dephosphorylation of occludin, and rearrangement of tight junctions (63). Lithocholic acid (LCA), on the other hand, can downregulate the expression of tight junction proteins and genes in IPEC-J2 cells (64) and induce apoptosis in IPEC-J2 cells by activating CD95 clusters and caspase 8 (65), suggesting a detrimental effect of LCA on the intestinal barrier (66). The receptors TGR5 and FXR for BAs have also been identified as crucial in the regulation of intestinal barrier function. Tauroursodeoxycholic acid (TUDCA) has been shown to ameliorate epithelial barrier damage induced by Escherichia coli in IPEC-J2 cells through TGR5 activation (52) and to reverse the decrease in mRNA expression of ZO-1 (zonula occludens 1), JAM, occludin, and claudin-4 in the mouse intestine (66). FXR in the ileum regulates Angiopoietins 1, Inducible Nitric Oxide Synthase, and Interleukin-18, ensuring adequate intestinal protection during periods of heightened microbial exposure while preventing excessive protein production that could lead to inflammation and intestinal diseases (40).

### 4.2 Mucosal barrier

The intestinal epithelium is covered by a mucus layer secreted by goblet cells, which comprises water, phospholipids, mucins (MUC), secreted immunoglobulin A (IgA), antimicrobial peptides, and various defense factors (54). This mucus layer plays a crucial role in preventing the adhesion and invasion of microorganisms and harmful substances, shielding epithelial cells from physical abrasion, and facilitating epithelial renewal and differentiation (67, 68). The mucus layer is structured into two distinct layers: an inner mucus layer that is tightly bound to the epithelial cells, creating a barrier that prevents bacterial penetration and maintains a sterile environment at the epithelial surface, and an outer mucus layer that is more loosely associated and provides a habitat for gut microbiota (69, 70). Notably, the inner mucus layer in the small intestine is thinner than that in the large intestine, likely to optimize nutrient absorption (71). Mucin, the primary component of the mucus layer, is essential for protecting the intestine from microbial infections (72). The human mucin family consists of 24 members (MUC1 to MUC24), categorized into secreted mucins and transmembrane mucins. Transmembrane mucins include MUC1, MUC3A/B, MUC4, MUC13, MUC15-17, MUC20, and MUC21, while secreted mucins encompass MUC2, MUC5AC/B, MUC6, MUC7, and MUC19 (73). Impairments in mucin production can compromise intestinal barrier function and contribute to the development of intestinal-related diseases, such as inflammatory bowel disease (IBD), irritable bowel syndrome (IBS), and cancer (56).

The mucus produced by goblet cells, particularly the mucin component, is crucial for protecting the intestinal mucosa (74, 75). The secretion of mucus is regulated by endoplasmic reticulum stress and autophagy. TUDCA has been shown to alleviate endoplasmic reticulum stress, stimulate the secretion of mucus from goblet cells, increase the thickness of mucus layers, and bolster the protective function of chemical barriers (76). Chenodeoxycholic acid (CDCA) activates FXR in normal rat gastric epithelial cells in a dose-dependent manner, leading to an upregulation of MUC2 protein expression and an enhancement of the chemical barrier (77). Research indicates that BAs can activate the FXR/nuclear transcription factor-κB (NF-κB) signaling pathway, which contributes to the promotion of MUC2 expression (78). Conversely, BAs can also compromise chemical barriers; for instance, deoxycholic acid (DCA) induces endoplasmic reticulum stress in the intestinal mucosa *in vitro* and exerts toxic effects on goblet cells, resulting in damage and a diminished protective capacity of chemical barriers (79). Additionally, bile acid receptors are significant in maintaining chemical barriers. Evidence suggests that the knockout or inhibition of the bile acid re-ceptor FXR leads to a reduction in MUC2 levels in mouse intestinal organoids, but the application of the FXR agonist GW4064 to activate FXR in mice has been found to mitigate radiation Ninduced intestinal damage (80). However, the specific knockout of FXR in the mouse liver appears to enhance colonic mucosal barrier function (81).

### 4.3 Microbial barriers

The gut microbial community comprises bacteria, fungi, viruses, archaea, and protozoa, with bacteria constituting over 70% of this population (82, 83). The majority of gut bacteria belong to two primary phyla: Bacteroidetes and Firmicutes (84). These intestinal bacteria play a crucial role in enhancing the host’s digestive efficiency by breaking down dietary polysaccharides into absorbable compounds, such as short-chain fatty acids (85). Additionally, they provide protection against pathogenic infections. Firstly, pathogenic bacteria face competition for nutrients from the gut microbiota, which restricts their ability to colonize the gut (86, 87). Secondly, the gut microbiota can stimulate the host’s immune response, thereby preventing pathogen invasion. For instance, the immune response triggered by symbiotic bacteria activating epithelial Toll-like receptors (TLRs) limits the proliferation of Salmonella typhimurium serotypes (88). In sterile mice, a reduced proliferation rate of intestinal epithelial cells, shorter crypt-villus length, and diminished angiogenesis have been observed; however, short-term supplementation with specific bacteria has demonstrated a proliferative effect in these mice, suggesting that a normal microbiota can stimulate cryptosystem cell activity, thereby influencing the proliferation rates in the colon and small intestine (89). Furthermore, the microbiota contributes to the integrity of the epithelial barrier. For example, butyrate produced by intestinal bacteria promotes wound healing by enhancing oxygen utilization, boosting anaerobic conditions, and activating compensatory hypoxia inducible factors (HIF), ultimately improving barrier function (90). Additionally, deoxycholic acid (DCA) generated by bacteria can facilitate mucosal wound healing by modulating local Prostaglandin E2 levels (91).

Primary BAs are transformed into secondary BAs by intestinal microorganisms. While these microorganisms metabolize BAs, the BAs simultaneously influence the gut microbiota (92). CA can inhibit bacterial growth by causing membrane damage. As the concentration of CA increases, the internal pH level of the bacteria gradually decreases, ultimately leading to the complete dissipation of both pH and transmembrane potential. Furthermore, potassium ion leakage was observed when the CA concentration exceeded 2 mM, and an increase in the leakage of other cellular components was noted when the CA concentration surpassed 4 mM (93). Additionally, bile acid salts that penetrate bacterial cells may inflict damage on bacterial nucleic acids, triggering SOS responses and causing oxidative damage to DNA, thereby hindering bacterial reproduction (94, 95). The antibacterial efficacy of various BAs differs, with bound BAs exhibiting lower antibacterial activity compared to their unbound counter-parts (96). This discrepancy may arise from the ability of unbound BAs to passively diffuse across membranes, resulting in intracellular toxicity, whereas bound BAs are fully ionized at physiological pH and only exert toxicity when specific transport proteins facilitate their entry into the cell (97). Furthermore, bacterial tolerance to BAs varies among species. The distinct structural characteristics of cell walls in Gram-negative bacteria allow them to exclude antibiotics and other antibacterial agents, potentially resulting in greater resistance to BAs (98). In contrast, Gram-positive bacteria tend to be more susceptible to bile action (97). For instance, Gram-negative bacteria such as Salmonella and Escherichia coli can thrive in the gallbladder, where bile concentrations are extremely high (99), while the growth of Lactobacillus acidophilus, a Gram-positive bacterium, is significantly inhibited in the presence of BAs (100).

### 4.4 Immune barrier

The mucosa associated lymphoid tissue (MALT) located in the intestinal lamina propria represents the largest immune organ in the human body, playing a pivotal role in the intestinal immune response (101). MALT is primarily divided into two components: the induction site and the effector site. The induction site comprises Peyer’s patches (PP), mesenteric lymph nodes (MLN), and isolated lymphoid follicles (ILF), which are crucial for the initial activation and differentiation of immune cells (102). The effector sites include the intestinal lamina propria and epithelium, which are essential for maintaining the integrity of immune cells and barriers (103). Immune cells are categorized into innate immune cells (such as innate lymphocytes, dendritic cells (DCs), macrophages, and natural killer cells) and adaptive immune cells (including B and T cells) (104). DCs and macrophages play a key role in recognizing antigens and presenting them to lymph nodes, thereby facilitating the generation of specific T and B cells. These specific T and B cells subsequently migrate back to the mucosal lamina propria to act as effector cells or to persist as memory cells (105). T cells are involved in cellular immunity, while immunoglobulin A (IgA) secreted by B cells serves as the primary antibody in the intestine, predominantly in the form of Secretory Immunoglobulin A (SIgA). SIgA functions by obstructing the entry of antigens, microorganisms, and exogenous proteins at the intestinal surface (102). Typically, SIgA is produced in response to microbial stimulation (103) and is present throughout the entire intestine (106). Upon re-infection, the titer of IgA antibodies rises significantly more rapidly and remains elevated for an extended duration, thereby enhancing the body’s ability to combat infections (102).

Bile acids play a crucial role in regulating intestinal mucosal homeostasis and the inflammatory response through various receptors, including FXR and TGR5, along with their associated signaling pathways (107). Studies have demonstrated that treatment with INT-747, an FXR agonist, in mice with colitis results in a reduction of pro-inflammatory cytokines such as Interleukin-1 beta (IL-1β) and Interleukin-6 (IL-6), as well as chemo-kines like C-C motif ligand 2 (41). Activation of FXR inhibits NF-κB activity by preventing the clearance of nuclear helper receptors at the binding sites for tumor necrosis factor (TNF) and IL-1β (108). *In vitro* studies indicate that FXR activation can enhance the release of proinflammatory cytokines, while Caco-2 cells treated with FXR antagonists show a significant decrease in the secretion of IL-6 and TNF (109). *In vivo*, FXR activation has been associated with reduced levels of TNF in models of dextran sulfate sodium (DSS) colitis (110). Furthermore, the knockout of the FXR receptor alleviates intestinal barrier dysfunction induced by lipopolysaccharide (LPS) damage and mitigates inflammatory injury. This protective effect is attributed to the decreased production of inflammatory cytokines, which aids in maintaining the integrity of tight junctions (111). Additionally, BAs can promote the differentiation of monocytes into DCs that secrete low levels of IL-12 and TNF-α via the TGR5-cAMP pathway (112), while also inhibiting the activation of the NOD-like receptor thermal protein domain associated protein 3 (NLRP3) inflammasome through the TGR5-cAMP-PKA axis (113), underscoring the significant role of BAs in modulating inflammatory responses. Research has found that the levels of pro-inflammatory cytokines (IL-1 β and TNF α) in the colon of low birth weight (LBW) animals are elevated and UDCA is significantly reduced. However, after supplementing UDCA, UDCA can induce M2 polarization of macrophages, inhibit NF - κ B, and exert anti-inflammatory effects in the intestine (114). BAs can also regulate the differentiation of T lymphocytes, which to some extent affects the homeostasis of the intestinal immune barrier. T helper cells expressing interleukin-17A (Th17 cells) help resist extracellular pathogens, while secondary BAs isodeoxycholic acid (isoDCA) can inhibit Th17 cell differentiation by suppressing ROR γ t (retinal acid receptor related nuclear receptor γ t), which may be closely related to IBD (115).

In general, the intestine serves not only as a crucial digestive organ but also functions as a barrier that separates the luminal contents from the body’s internal environment through the intestinal mucosa. Acting as a physical, biochemical, and immune barrier, it engages with the external environment to safeguard the internal milieu from harmful substances. While certain BAs may exert detrimental effects on the gut, they are vital for preserving the integrity of the gut barrier. BAs play a significant role in cell proliferation and apoptosis, regulate both mucosal and mechanical barriers, inhibit the growth of specific harmful bacteria, and are involved in the immune response within the gut.

## 5 Conclusion

Since the identification of the bile acid receptor FXR, there has been a growing interest in the role of BAs as signaling molecules that influence cell growth and immune responses. The interaction between BAs and their key receptors, FXR and TGR5, along with the intestinal barrier, is crucial for maintaining intestinal barrier integrity. This article reviews the functions of bile acid metabolism and the intestinal barrier, detailing the regulatory mechanisms by which BAs and their primary receptors, FXR and TGR5, influence the mechanical, mucosal, microbial, and immune barriers of the intestine. This underscores the significant role of BAs and their receptors in preserving intestinal barrier homeostasis. Nonetheless, further investigation is needed to enhance our understanding of the intricate relationship between BAs and the intestinal barrier, clarify their potential mechanisms of action, and identify associated risks, ultimately aiming to develop more effective treatment strategies for intestinal diseases.
